# Analgesic effect of epidural anesthesia via the intervertebral foramen approach in percutaneous transforaminal endoscopic discectomy: a retrospective study

**DOI:** 10.1186/s12871-022-01924-x

**Published:** 2022-12-20

**Authors:** Jingyue Zhang, Xueyao Wang, Zhenhua Cai, Jiyu Kang, Yongliang Liu, Chunyan Nie, Huacheng Zhou

**Affiliations:** 1 Department of Pain Management, the Fourth Affiliated Hospital of Harbin Medical University, No.37, Yiyuan Street, Nangang District, Harbin, 150001 Heilongjiang Province China; 2 Department of Pain Management, the First Affiliated Hospital of Harbin Medical University, No.25 Post Office Street, Nangang District, Harbin, 150001 Heilongjiang Province China; 3 Department of Pain Management, the Second Affiliated Hospital of Harbin Medical University, No.246, Xuefu Street, Nangang District, Harbin, 150001 Heilongjiang Province China

**Keywords:** Local anesthesia, Epidural anesthesia, Percutaneous transforaminal endoscopic, Neurological complications

## Abstract

**Background:**

Satisfactory intraoperative analgesia is critical for percutaneous transforaminal endoscopic discectomy (PTED). Local anesthesia (LA) and epidural anesthesia (EA) are recommended for PTED. LA alone does not achieve satisfactory pain management during PTED and other analgesics or sedatives are usually needed. Traditional EA, which involves implanting an epidural catheter through the midline or paramedian, has disadvantages such as difficulty in catheterization and increased preoperative preparation time. Rather than performing conventional EA, we injected local anesthetics through the intervertebral foramen during the puncture process, which we termed lumbar transforaminal EA (LTEA), and observed its feasibility and safety. This study aimed to conduct a comprehensive comparison of differences in analgesia between LA and LTEA in patients with PTED.

**Methods:**

We performed a retrospective analysis of patients who underwent PTED between January 2018 and January 2021. Patients were divided into LA and LTEA groups. Data obtained from the electronic medical records included primary outcomes (visual analog scale [VAS] scores and anesthesia satisfaction rate) and secondary outcomes, including vital signs such as heart rate (HR), mean arterial pressure (MAP), total dosage of fentanyl, operation time, X-ray exposure time, Oswestry Disability Index (ODI) scores, and complications.

**Results:**

In total, 160 patients (80 in each group) were analyzed in this study. The VAS scores for lumbar and leg pain were significantly lower in the LTEA group than in the LA group (*P* < 0.0001). The anesthesia satisfaction rate was 90.0% in the LTEA group and 72.5% in the LA group (*P* < 0.005). MAP and HR values in the LTEA group were significantly lower than those in the LA group (*P* < 0.05). The total dose of fentanyl in the LTEA group was significantly lower than that in the LA group (*P* < 0.05). As for ODI values, the average operation time, X-ray exposure time, and incidence of complications were not significantly different between the two groups (*P* > 0.05).

**Conclusions:**

LTEA simplifies the process of EA and can achieve a good analgesic effect intraoperatively without increasing the preoperative preparation time; thus, it may be adopted as an alternative mode of anesthesia during PTED surgery.

## Introduction

Intervertebral disc herniation (IDH) is a spinal condition that can causes low back pain (LBP) and/or radiculopathy and accounts for a large proportion of patients undergoing spine surgery annually [1, 2]. Approximately 40% of LBP is caused by intervertebral disk degeneration, which also commonly causes IDH, especially via its excessive load effects on degenerated intervertebral discs [3–5]. Other clinical symptoms of IDH include sciatica, numbness, and amyotrophy of the lower limbs [6]. Minimally invasive surgery, especially percutaneous transforaminal endoscopic discectomy (PTED), has grown in popularity in recent years when physical therapy, drugs, nerve blocks, and other nonsurgical treatments fail [7, 8]. Compared with open surgeries, PTED showed more favorable outcomes for self-reported leg pain, back pain, functional status, quality of life, and recovery [9]. Currently, the indications for PTED have expanded from pure IDH to spinal stenosis, lumbar metastatic tumors, adjacent segmental degeneration after lumbar fusion, and revision of recurrent IDH [10, 11].

Satisfactory intraoperative analgesia is critical in PTED. PTED is performed around the nerve roots; thus, awareness and certain motor functions of patients must be maintained to minimize the risk of nerve root injury. General anesthesia (GA) provides complete analgesia and sedation; however, GA for PTED may lead to a greater risk of neurological complications owing to patients' inability to perceive and respond to nerve nociceptive stimulation [12–14]. Local anesthesia (LA) is recommended in clinics to avoid nerve injury; however, many surgeons have found that several patients cannot tolerate the pain during surgery, especially during the process of foraminoplasty and repair of the annulus fibrosus and posterior longitudinal ligament [12].

Epidural anesthesia (EA), which involves implantation of an epidural catheter through the midline or paramedian, is another method that can keep patients awake during surgery and provide a better analgesic effect than LA [8, 13]. However, traditional EA has disadvantages such as difficulty in catheterization, possibility of nerve root damage, abnormal drug distribution, and increased preoperative preparation time.

Rather than performing conventional EA, we injected local anesthetics through the intervertebral foramen during the puncture process, a process that we termed lumbar transforaminal EA (LTEA). In clinical practice, we found that this method worked effectively against analgesia. Nerve blocking through the LTEA approach has been found to be more effective than conventional treatment in reducing pain and improving dysfunction in patients with LBP and radicular pain [15–17]. Although LTEA is a common and mature technology, it has rarely been used for intraoperative analgesia in PTED. In this retrospective analysis, we conducted a comprehensive comparison of the differences in analgesia between LA and LTEA in PTED patients and assessed the efficacy and safety of LTEA on pain relief in patients who underwent LBP with radicular pain and who received PTED.

## Methods

### Patients

This was a retrospective observational study of patients with IDH who underwent PTED; it was conducted from January 2018 to January 2021 at the Fourth Hospital Affiliated Harbin Medical University. The study protocol was approved by the local ethics committee (Protocol No: 2022-SCILLSC-12), and the study was conducted in accordance with the Declaration of Helsinki and its later amendments.

**Fig. 1:**
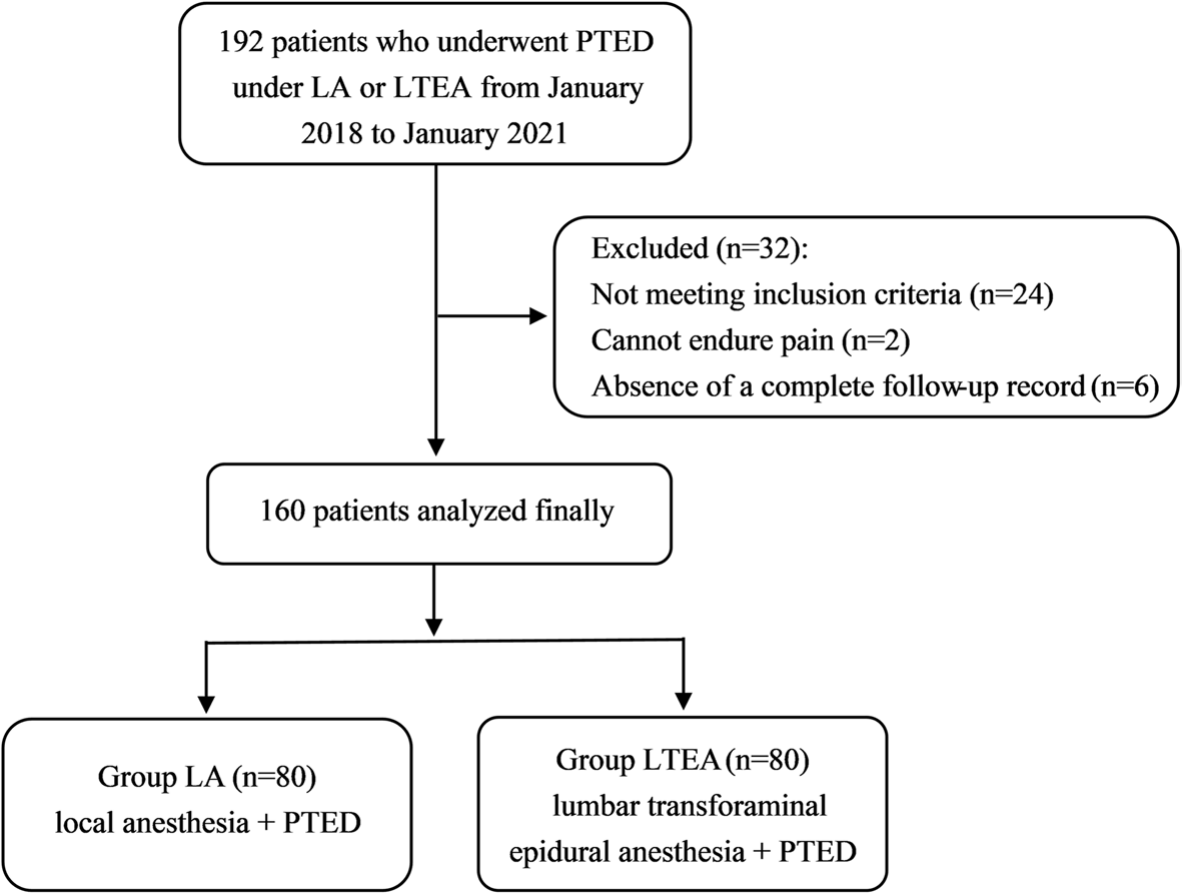
Study flow chart. PTED: percutaneous transforaminal endoscopic discectomy; LA: local anesthesia; LTEA: lumbar transforaminal epidural anesthesia

The inclusion criteria were as follows: (1) American Society of Anesthesiologists grade I or II, (2) imaging demonstrating a single lumbar disc herniation, (3) typical symptoms of nerve compression, (4) corroborative clinical and radiological findings, and (5) PTED either under LA or LTEA. The exclusion criteria were as follows: (1) severe systemic disease, (2) multisegmental lumbar disc herniation, (3) clotting disorder, (4) spinal deformity, and (5) absence of a complete follow-up record. A total of 192 patients who underwent PTED strictly implemented LA or LTEA method between Jan 2018–2021. After excluding 32 patients, the remaining 160 patients were analyzed in this study. According to different anesthesia methods, 80 patients were under local anesthesia and enrolled in LA group; 80 patients were under lumbar transforaminal EA and enrolled in LTEA group (Fig. 1). Informed consent was obtained from all patients.

### Surgical procedure

All patients were monitored for vital signs after entering the operating room and inhaled 2 L/min oxygen with a nasal catheter. Midazolam (0.02 mg/kg, (Yichang Renfu pharmaceutical Co. LTD, Hubei, China) and fentanyl (1 µg/kg, Yichang Renfu pharmaceutical Co. LTD, Hubei, China), as well as metoclopramide (10 mg, Wuxi Seventh Pharmaceutical Co. LTD, Jiangsu, China) were injected intravenously for each patient. All Patients were placed in a lateral position with their affected side on top. The puncture track was determined using a C-arm and K-wire, and the insertion site was marked. In the LA group, after infiltration of the skin, an acupuncture guide needle was used to anesthetize the trajectory layer-by-layer with a local anesthetic mixture (0.5% lidocaine + 0.125% ropivacaine). After the needle reached the superior articular process (SAP), 15 ml of contrast media plus anesthetic was injected to infiltrate the SAP. In the LTEA group, after infiltration of the skin, 5 ml of local anesthetic was injected to infiltrate the SAP; then, the needle was moved across the upper edge of the SAP and moved forward until the needle tip was in the posterior 1/3 to 1/2 of the neural foramen in the lateral view, under the pedicle (6 o’clock position) in the AP view, and until resistance disappeared. Then, a 10-ml mixture of contrast agent and local anesthetic was injected. The distribution of the contrast agent was equivalent to the actual anesthetic distribution.

After anesthesia, the needle was withdrawn to the leading edge of the SAP, a guidewire was inserted into the tissue, the surgeon cut the skin with a 0.7-cm incision along the guidewire, the dilator tube was inserted, the SAP was polished with a ring saw, and part of the SAP was removed to enlarge the intervertebral foramen. Next, a working cannula was inserted, and the final target position of the working cannula under radiological guidance was at the same position as the tip of the needle. The anteroposterior view was between the spinous process and vertebral pedicle, and the lateral view was at the posterior edge of the disc plane. If the herniated disc upturned or if sagging free disk was involved, the cannula tip was placed at the midpoint to the end of the protrusion and at the edge of the vertebral body. Finally, an endoscope was connected, and the herniated disc was removed using endoscopic nucleus pulposus forceps. Once there was sufficient space around the nerve root, the endoscope was removed, and the skin was sutured. Fentanyl was administered to patients in case of unbearable pain during the operation (VAS score ≥ 4).

### Clinical evaluation

The clinical parameters of all patients were obtained from their medical records. The primary outcomes included assessment of pain, where a 10-cm visual analog scale (VAS, in which 0 cm represented no pain, and 10 cm the worst imaginable pain) [18] was used to assess lumbar and leg pain at the following time points: pre-operation (T0), during the working cannula placement phase (T1), during the partial SAP removal phase (T2), during the nucleus pulposus removal phase (T3), during the incitement of the posterior longitudinal ligament and posterior margin of the AF (T4), at the end of the operation (T5), 1 week postoperatively (T6), and 1 month postoperatively (T7). Anesthesia satisfaction rate was assessed using a 5-point Likert-type scale [19] criteria at the end of the operation (T5), with a score of 0 indicating a very bad experience and a score of 5 indicating a very good experience. The satisfaction rate was calculated as the number of patients rated who rated their experience as “very good” and “good” divided by the total number of patients in each group.

The secondary outcomes were as follows: (1) vital signs including heart rate (HR) and mean arterial pressure (MAP). Preoperative and intra-operational changes (T0-T5) in HR and MAP were analyzed; (2) operation time, considered from the time of LA administration to incision suturing; (3) X-ray exposure time; (4) total dosage of fentanyl used intraoperatively; (5) complications such as nausea, vomiting, dizziness, drowsiness, local anesthetic intoxication, lower limb weakness, subarachnoid anesthesia, and urinary retention; and (6) disability status of the enrolled patients, assessed using the Oswestry Disability Index (ODI) [20] at baseline, 1-week and 1-month postoperatively (T0, T6, T7). In addition, demographics, including sex, age, height, and weight, were analyzed between the two groups.

### Statistical analysis

SPSS 22.0 (IBM Corporation, NY, USA) was used for the statistical analysis. Height, weight, VAS, HR, MAP, operation time, and total dosage of fentanyl were represented by the mean ± standard deviation values and were compared using the independent two-sample *t*-test. Anesthesia satisfaction rate, incidence of complications, and sex and age were compared using the chi-square test. Repeated measurement data were analyzed using repeated-measures measurement ANOVA. *P* < 0.05 was considered statistically significant.

## Results

**Fig. 2:**
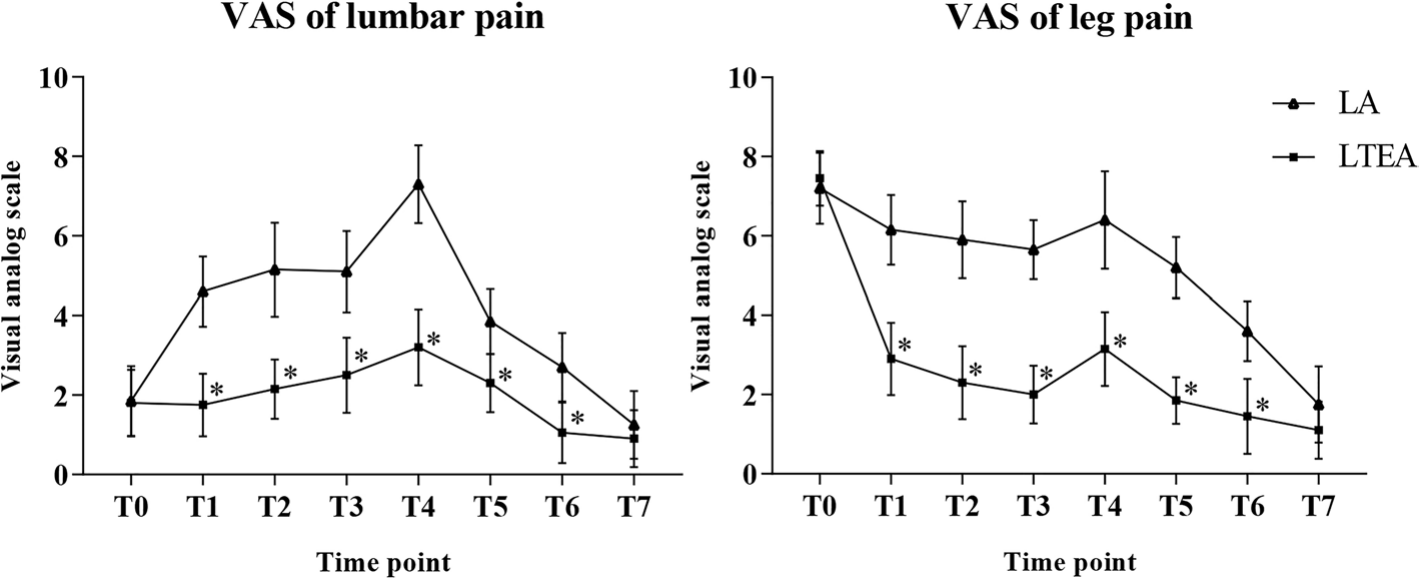
VAS score in the LA group and LTEA group at different time points. VAS, Visual analog scale; LA, local anesthesia; LTEA, lumbar transforaminal epidural anesthesia. *P < 0.0001

There were no statistically significant differences in the demographics (Table 1). With respect to the primary outcome (Fig. 2), there was no obvious difference between the two groups in terms of lumbar and leg pain at baseline (at time point T0, *P* > 0.05). However, VAS scores of the lumbar pain were significantly lower in the LTEA group than in the LA group during the operation (at T1–T5, *P* < 0.05) and 1-week post-operation (T6). No significant differences were evident at 1-month post-operation (T7). As for leg pain evaluated using VAS scores, no statistical difference was detected between the two groups at baseline and 1-month post-operation (T0 and T7). Nevertheless, during the operation and at 1-week post-operation (T1–T6), significant differences were detected between the two groups, and the VAS scores for leg pain were lower in the LTEA group than in the LA group (*P* < 0.001). In summary, the results revealed significant intergroup differences in intraoperative (*P* < 0.001) lumbar and leg VAS scores, with LTEA being superior to LA in intraoperative pain relief. No intergroup differences were observed in the 1-month postoperative (*P* < 0.001) lumbar and leg VAS scores, with the LTEA group’s results appearing similar to the LA group’s results.

**Table 1:** Patient demographic characteristics between two groups

	LA group (n = 80)	LTEA group (n = 80)	P
**Sex**			
Male	53	49	0.51
Female	27	31	
**Age**	66.0 ± 10.1	67.3 ± 10.5	0.69
**BMI (kg/m**^**2**^**)**	23.7 ± 1.8	24.3 ± 2.3	0.53
**Segment**			
L2-3	5	5	
L3-4	13	11	
L4-5	37	42	
L5-S1	25	22	
**Type of lumbar disc herniation**			
Central	12	8	
Paracentral	60	66	
Foraminal	6	5	
Extremely	2	1	

BMI, body mass index; LA, local anesthesia; LTEA, lumbar transforaminal epidural anesthesia

Regarding the anesthesia satisfaction rate at the end of the operation (T5), we observed that the patient satisfaction rate in the LTEA group was significantly greater than that of the LA group (90.0% *vs*. 72.5%, *P* < 0.05). A total of 27.5% of patients in the LA group and 10.0% the LTEA group were dissatisfied with the anesthesia. Patients in the LA group complained of pain or could not tolerate the pain during the herniation discectomy and repair of the annulus fibrosus and posterior longitudinal ligament. None of the patients in the LTEA group complained of pain during the surgery (Table 2).

**Table 2:** Comparison of the clinical outcomes between two groups

	LA group (n = 80)	LTEA group (n = 80)	P
**Operative time(minutes)**	73.9 ± 6.0	78.6 ± 4.8	0.07
**X-ray exposure time(seconds)**	28.8 ± 1.9	26.9 ± 2.7	0.08
**Dosage of fentanyl(μg)**	37.5 ± 26.3	17.5 ± 21.6	0.01^*^
**Experience with anesthesia** **(5-point Likert-type scale)**			
Very bad	5	0	
Bad	7	3	
Neutral	10	5	
Good	28	34	
Very good	30	38	
**Satisfaction rate of anesthesia**	58(72.5%)	72(90%)	0.01^*^
**Complications**	5(6.25%)	6(7.5%)	0.76
Nausea	1	1	
Vomiting	1	0	
Nerve injury	0	0	
Local anesthetic intoxication	1	0	
Limb weakness or transient paresis	2	4	
Subarachnoid anesthesia	0	1	
Urinary retention	0	0	

LA, local anesthesia; LTEA, lumbar transforaminal epidural anesthesia; ^*^P < 0.05, the difference was statistically significant

**Fig. 3:**
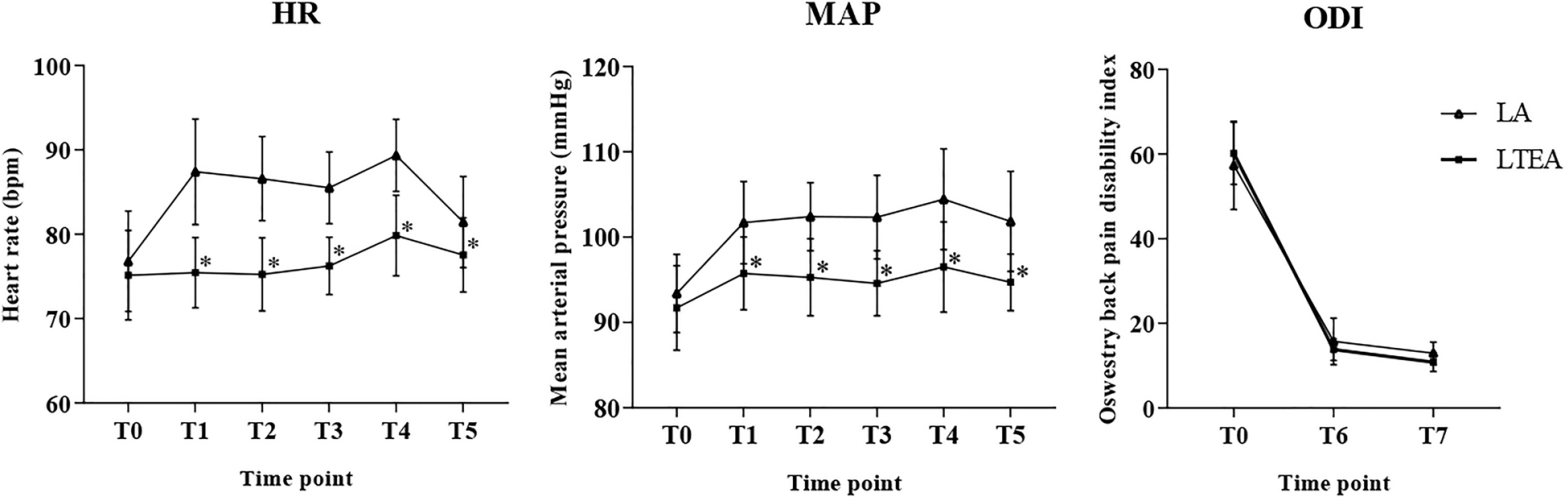
Heart rate, mean arterial pressure and ODI in two groups at different time points. HR, heart rate; MAP, mean arterial pressure; ODI, Oswestry disability index. * *P* < 0.05

With respect to the secondary outcomes at the baseline (T0), the HR and MAP values of the two groups showed no significant differences. However, HR and MAP values were significantly higher in the LA group than in the LTEA group during the operation (T1–T5, *P* < 0.05). Mean ODI values in both groups decreased significantly at 1-week and 1-month post-operation (T6, T7) compared to that at baseline (T0) (LA, ODI from 57.4 to 15.8, 13.0, *P* < 0.0001; LTEA, ODI from 60.2 to 13.8, 10.9, *P* < 0.0001, respectively). Nevertheless, there was no significant difference between the two groups at 1-week and 1-month post-operation (T6, T7) (*P* = 0.66 and *P* = 0.60, respectively) (Fig. 3). There were no significant differences in the operation times between the two groups (*P* = 0.07). The X-ray exposure time in the LTEA group was slightly shorter than that in the LA group, but the difference was not significant (*P* = 0.08) (Table 2). In terms of the application of analgesic drugs during the operation, the total dose of fentanyl in the LTEA group was lower than that in the LA group, and the difference was significant (*P* < 0.05) (Table 2).

**Fig. 4:**
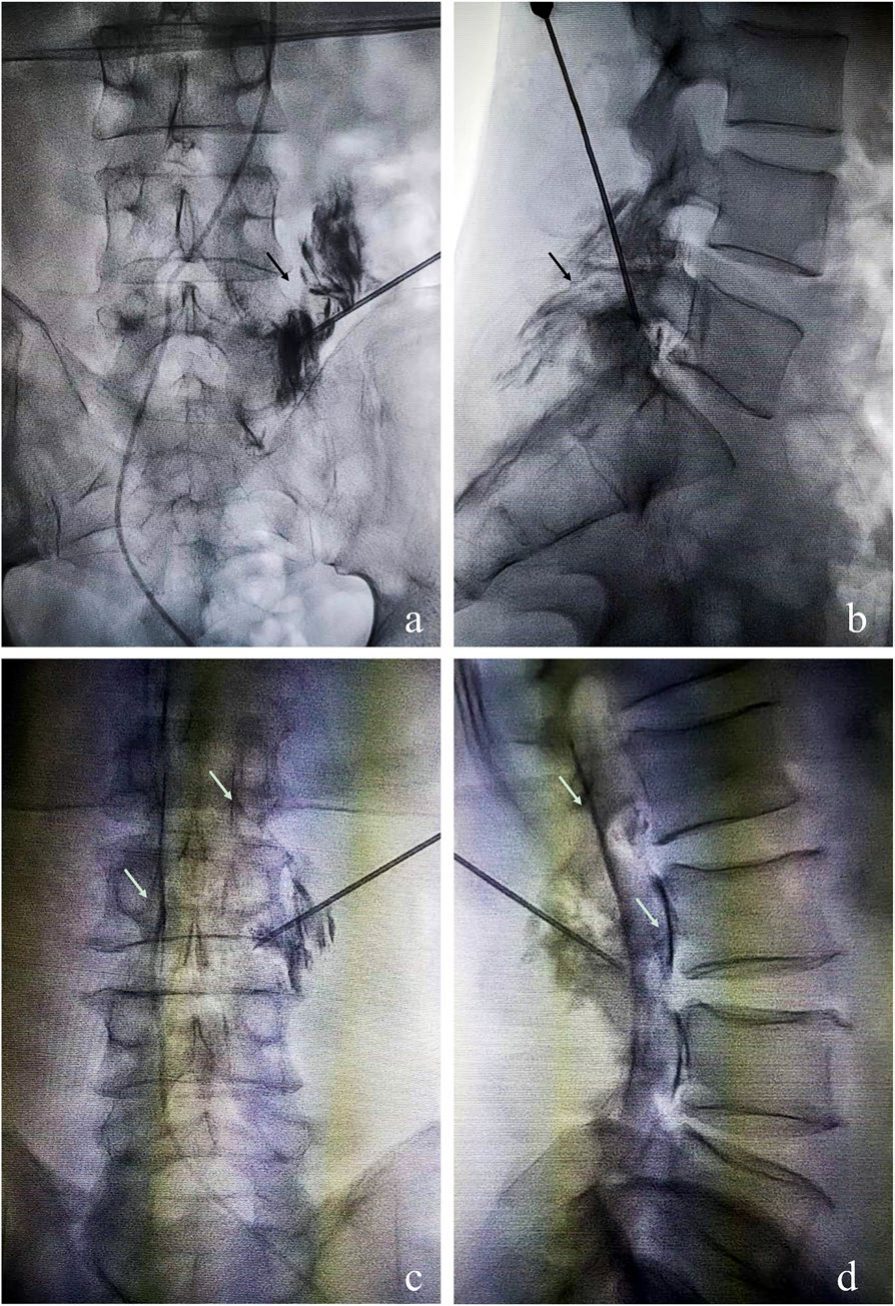
Anteroposterior and lateral intraoperative fluoroscopic images after contrast medium application using LA and LTEA. **a**, **b** Distribution of contrast media during LA. The black arrow indicates the distribution of the contrast media; **c**, **d** Distribution of the contrast media during LTEA. The white arrow indicates the double-track sign of the contrast media entering the epidural space

X-ray fluoroscopy was used to observe the contrast agent distribution in both groups. The results showed that in the LA group, the contrast agents were mainly distributed in the paravertebral area, and in the LTEA group, the contrast agents were mainly distributed in the spinal canal and paraspinal area (Fig. 4).

With regard to the incidence of complications in the LA group, there were two cases of transient leg paresis or weakness during surgery, accounting for 2.5% of patients in the group, as well as two cases of nausea and vomiting and one case of local anesthetic intoxication. With regard to the incidence of complications in the LTEA group, there were four cases of transient leg paresis or weakness during surgery, accounting for 5% of patients in the group, as well as one case of nausea and vomiting and one case of subarachnoid anesthesia. Patients in both groups had transient leg paresis or weakness caused by the spread of anesthetic drugs to the lumbar plexus during the administration of the superior articular process anesthesia. These patients recovered 3–4 days after surgery. No urinary retention was observed in either group. No significant difference was observed in the incidence of complications between the two groups (*P* = 0.76) (Table 2).

## Discussion

Patients in both groups completed the PTED procedure, and no neurological complications were observed. The data demonstrated that LTEA obviously reduced VAS scores for lumbar and leg pain during the operation, decreased the total dose of fentanyl, and achieved a better anesthesia satisfaction rate without causing significant fluctuations in vital signs compared to LA. In other words, patients can achieve better pain relief when the local anesthetic is distributed in the epidural space via the transforaminal approach. No serious complications occurred during the surgery.

LA has the advantage of being a simpler procedure with fewer anesthesia-related complications. However, the effectiveness of LA in managing intraoperative LBP and leg pain is unsatisfactory [12]. During PTED surgery, intraoperative LBP occurs mainly due to local nerve irritation caused by the incitement of the posterior longitudinal ligament and the posterior margin of the inflammatory annulus fibrosus [21]. Intraoperative leg pain is attributed to nerve root irritation during cannula placement and during the nucleus pulposus extraction procedure [22]. More specifically, LA is not effective in controlling the pain caused by nerve root traction, cannula placement, and incitation of the posterior longitudinal ligament [21, 23], with patients sometimes foregoing the procedure due to intolerable pain [14]. Thus, the necessary use of opioid analgesics is increased with LA, which increases adverse reactions such as nausea and vomiting [24]. On the other hand, moderate to severe pain can increase one’s heart rate and blood pressure, leading to a high surgical risk in patients with cardiovascular and cerebrovascular diseases [25]. In this study, owing to unsatisfactory intraoperative pain management, MAP and HR fluctuations occurred in the LA group during the intraoperative working cannula placement phase, partial SAP removal phase, and nucleus pulposus removal phase (time points T1, T2, and T3), and more fentanyl was administered to control pain. On the contrary, no significant fluctuations in the MAP and HR values were observed, and the dosage of fentanyl was lower in the LTEA group, which indicated that patients in the LTEA group received better pain management.

EA with low concentrations of ropivacaine leads to sensorimotor separation that blocks the sensory nerves and preserves motor function, allowing surgeons to receive immediate feedback from patients when the nerves are irritated [23]. However, the disadvantages of EA compared to LA are that the procedure is more complicated, the operation and postoperative bed times are prolonged, and the risk of anesthesia-related complications such as urinary retention is increased [12]. In this study, the postoperative anesthesia satisfaction survey showed that 90.0% of patients had a very good or good experience with the transforaminal EA, whereas only 72.5% of patients in the LA group had a very good or good experience. When patients can better tolerate discomfort during an operation, a higher anesthesia satisfaction rate can be achieved. The results once again demonstrate that transforaminal EA not only satisfies the requirement of keeping patients awake but also provides a good analgesic effect. Similar results have been found in other studies, with EA reported as being superior to LA for SAP [8, 12–14, 23]. ODI is used widely to evaluate the degree of functional dysfunction in patients with LBP [26]. In this study, there were no significant intergroup differences in postoperative ODI scores, indicating that LTEA did not cause any more dysfunction than LA in the waist region. The results indicated that both groups recovered well after surgery, which is consistent with the findings of previous studies [12, 13].

Epidural injections can be an effective treatment for radicular pain while also providing the potential for functional improvement. There are three main interventional approaches: interlaminar, transforaminal, and caudal. The risks and efficacy data vary between these routes of injection, and efficacy data for the underlying pathology of the transforaminal route are the most robust [27]. A transforaminal approach allows the drug to be injected directly into the anterior epidural space, closer to the nerve root. With interlaminar and caudal approaches, drug diffusion is often difficult near the affected nerve roots; thus, epidural injections through the foramina are more efficacious than the other two methods [28, 29]. A recent review by Carassiti et al. highlights that chronic LBP can be managed by injecting steroids through a transforaminal approach, which is an interesting parallelism with this study [30]. Another study indicated that the evidence for transforaminal lumbar epidural injections is level II-1 for short-term relief and level II-2 for long-term improvement in the management of lumbar nerve root and LBP [31]. In this study, we used transforaminal EA, a method that simplifies the traditional catheterization procedure and is more accurate when aided by imaging, thereby reducing the overall length of the operation. For LA, low concentrations of ropivacaine produce an excellent sensory–motor block separation effect, and lidocaine works very quickly; hence, we chose a mixture of the two as the anesthetic in this study.

The advantages of LTEA are as follows: (1) one-time anesthesia can meet surgical requirements without separate puncture and catheter placement procedures; (2) anesthesia is mainly administered on the lesion side, and local anesthetics are more likely to be distributed in the ventral epidural space; and (3) LTEA involves more accurate and less local anesthetics. In addition, the position of the needle tip can be observed on radiographic examination. However, with traditional EA catheterization, the direction and position of the catheter vary greatly, which may affect the anesthetic effect during surgery.

GA has the advantage of providing complete analgesia and sedation during an operation; however, as patients may decide not to undergo GA due to the high incidence of neurological complications and high medical costs, it was not recommended in PTED [14]. In addition, choking, nausea, and vomiting after GA can increase negative pressure, which may induce the nucleus pulposus to protrude again. In this study, in the LTEA group, the local anesthetic diffused into the epidural space and resulted in a wider range of sensory nerve blockades for better pain management, which is consistent with previous research [8, 12, 13]. In addition, the protrusions of the lumbar disc herniations were mostly located in the ventral side of the dural sac and nerve roots. During transforaminal EA, local anesthetics can be injected into the anterior epidural space and around the nerve root sheath, which is closer to the lesion, in order to reduce pain during an operation.

During incision of the posterior longitudinal ligament, the VAS score for peak pain in the LTEA group was significantly lower than that in the LA group, indicating that transforaminal EA can effectively block the sensory nerve endings that are distributed in the posterior longitudinal ligament and foramina. In addition, we chose the same working cannula route of insertion for the administration of the transforaminal EA, which can reduce the time required to perform EA. The results demonstrate that transforaminal EA does not prolong the operative time compared to LA. In addition, the surgery was not interrupted by patients in the LTEA group as their intraoperative pain was minimal, which also led to less X-ray exposure time.

During the surgery, we observed some adverse reactions. One patient experienced intoxication during LA in the SAP. Excessive local anesthetic may lead to intoxication through bone surface absorption. We also observed several cases of transient lower limb weakness in the LTEA group, with patients unable to flex and extend their knee and ankle joints. The entry of local anesthetics into the lumbar plexus was considered the cause of this. In addition, there was one case of subarachnoid anesthesia in the LTEA group; however, the patient’s vital signs were stable, and the surgery was completed successfully. This was possibly due to the needle puncturing the nerve root sleeve and the drug partially entering the subarachnoid space. In light of the above, during the implementation of transforaminal EA, the ratio and dose of local anesthetics should be determined according to different populations, and the puncture needle tip should be precisely located to avoid complications.

Our study has some limitations. First, this study was a single-center clinical trial with a small sample size, which might reduce the power of our statistical results. Second, the current study was a retrospective study, and the lack of randomization and blinding may lead to biases. Third, we did not conduct hierarchical statistics based on age, sex, and other factors. Finally, there was no comparison with classical EA using the interlaminar approach. Next, we will design a prospective controlled study to investigate the different analgesic effect of transforming epidural with conventional epidural.

## Conclusions

In summary, LTEA simplifies the process of EA and can achieve a good analgesic effect intraoperatively without increasing the preoperative preparation time; thus, it may be adopted as an alternative mode of anesthesia during PTED surgery.

## Data Availability

The datasets used during the current study are available from the corresponding author on reasonable request.

## Abbreviations

PTED: Percutaneous transforaminal endoscopic discectomy
LA: Local anesthesia
EA: Epidural anesthesia
LTEA: Lumbar transforaminal epidural anesthesia
VAS: Visual analog scale
HR: Heart rate
MAP: Mean arterial pressure
ODI: Oswestry Disability Index
IDH: Intervertebral disc herniation
LBP: Low back pain
GA: General anesthesia
SAP: Superior articular process
BMI: Body mass index
